# Safety of Permanent Pacemaker Implantation: A Prospective Study

**DOI:** 10.3390/jcm8010035

**Published:** 2019-01-01

**Authors:** Mª Reyes Carrión-Camacho, Ignacio Marín-León, José Manuel Molina-Doñoro, José Rafael González-López

**Affiliations:** 1 Nursing Department, Faculty of Nursing, Physiotherapy and Podiatry, Universidad de Sevilla, 41009 Seville, Spain; joserafael@us.es; 2 Clinical Management Unit-Anesthesiology and Resuscitation, Cardiac Surgery, Virgen del Rocío University Hospital, 41013 Seville, Spain; 3 Internal Medicine, Virgen del Rocío University Hospital, IBIS-CIBERESP, Fundación Enebro, 41013 Seville, Spain; palomanacho@gmail.com; 4 Emergency Medicine, Virgen de Valme University Hospital, 41014 Seville, Spain; jmanuel.molina.sspa@juntadeandalucia.es

**Keywords:** pacemaker, artificial, anticoagulant drugs, postoperative complications, patient safety, prospective studies, outcome assessment

## Abstract

Although pacemaker implantation is considered to be low risk, it is not exempt from complications and technical failures during the procedure, both in the short and long term, and the complications that such patients may present remain unknown. The aim has been to analyze the complication rates associated with permanent pacing and to identify if these differ between patients with or without previous antithrombotic therapy. We used a prospective, single center, observational study of 310 adult patients with indications of permanent pacing. They were hospitalized from 1 January to 31 December 2014 and followed up for 6 months after the pacemaker implant. The participants were distributed into two groups according to the antithrombotic therapy prior to the implant. The most frequent major complications were pneumothorax (3.87%) and lead dislodgement (8.39%), while superficial phlebitis (12.90%) and uncomplicated hematomas (22.58%) were presented as the most recurrent minor complications. Hematomas were the most frequent minor complication in the antithrombotic therapy cohort, and shoulder pain was reported as the most recurrent minor complication in the non-exposed group. Finding out about complications in pacemaker implants enables a complete view of the process, and hence the prioritization of actions aimed at improving safety and reducing associated risks.

## 1. Introduction

Cardiac stimulation has become the common treatment of symptomatic bradycardia or high-grade atrioventricular block. Pacemaker implant rates have increased exponentially in the last few years, especially in the elderly [1]. The aging of the population, the technological advances of these devices, and the growing number of clinical indications are the main factors that contribute to the increase of this rate [2]. It is estimated that each year 1.25 million permanent pacemakers are implanted worldwide [1]. In 2016, approximately 500,000 permanent pacemakers were implanted in Europe and there were 37,466 implants in Spain [1]. The 2016 annual report of the Virgen del Rocío University Hospital indicates that 479 permanent pacemakers were implanted [3].

Although the procedure is considered to be minor surgery, this does not mean that it is exempt from complications and technical failures in the short and long term [4].

In this sense, the implant requires special consideration in patients with antithrombotic therapy, so perioperative management represents a challenge for the care needed by these patients [4]. The aim of our study was to prospectively identify the complication rates of patients after pacemaker implantation, according to a system of anticoagulant and antithrombotic treatment or without this.

## 2. Materials and Methods

### 2.1. Study Design and Participants

We proposed a prospective cohort study [5,6]. The sample was made up of patients with indications of permanent pacing, hospitalized in any of the Medical–Surgical Units or in the Critical Care and Emergency Unit of the Virgen del Rocío University Hospital in Seville (Spain) from 1 January to 31 December of 2014. The patients were followed up for 6 months after the pacemaker implant. This was done during the first 30 days, by telephone, with cut-off points at 7, 15, and 30 days. In the case of non-response, they were called again 48 h later to avoid losses during the follow-up procedure. A review of the clinical history was made after 6 months, exploring the presence of any episode related to the pacemaker implant documented as a complication.

Considering the aim of the study, all the cases were distributed into two groups depending on whether they had been prescribed antithrombotic therapy or not before the surgery.

### 2.2. Inclusion and Exclusion Criteria

In order to include a case in the study, it had to meet three requirements: (a) to be the first implantation of a permanent pacemaker, (b) the patient had to be older than 18 years old, and (c) the patient had to sign an informed consent to participate in the study. If patients had generator replacements, a device removal, or an implantation of defibrillators or resynchronizers, they were excluded from the sample.

Finally, the inclusion criteria were met by 310 patients.

### 2.3. Implant Procedure

Implant procedural aspects were defined as elements related to the preparation of the patient (antibiotic prophylaxis); aspects connected with the technique of the implant (difficulty of central venous access, use of support with image); data related to perioperative care (surgical wound compression, arm immobilization); elements related to patient follow-up; and with respect to the work team, data related to the surgeon’s experience (high >100 implants/year, medium <100 implants/year, and low <50 implants/year) [7,8]. All patients received compression dressing, local cold on the surgical wound, and an immobilization of the arm ipsilateral to the pacemaker implant before leaving the operating room.

### 2.4. Definition of Exposure

According to the treatment systems followed by the patient before the implantation, in terms of anticoagulant and antithrombotic treatment, and according to the protocol implemented in the hospital, the patients were distributed into two strata: (1) patients not treated with antithrombotic therapy, considered in the analysis as “not exposed” (*n* = 71; 23%); and (2) patients treated with anticoagulant and antithrombotic treatment, analyzed as “exposed group” (*n* = 239; 77%). This last group was subdivided into 4 different subgroups: (a) patients only with oral anticoagulation (*n* = 15); (b) patients with combination therapy consisting of oral anticoagulation/antiplatelet agent/bridging heparin (*n* = 103); (c) patients with simple or double antiplatelet therapy (*n* = 76); and (d) patients only with heparin (*n* = 45).

The anticoagulant treatment was Acenocumarol (10 patients) and new oral anticoagulants (5 patients). Regarding the antithrombotic therapy, the specific drugs were acetylsalicylic acid (61 patients) and Clopidogrel (9 patients). Finally, 148 patients took low molecular weight heparin.

### 2.5. Protocol for Discontinuation of Anticoagulant and Antithrombotic Treatment

The protocol implanted in our Hospital Center varied depending on the level of risk of thromboembolism. This was evaluated according to the PRETEMED guide [9] since it is one of the most used in Spain [10]. Therefore, the specific protocols are explained below:

(1) Patients with a mechanical heart valve, atrial fibrillation, or a high risk of thromboembolism are given bridging heparin (during 48 h after the procedure).
(2) Patients with a mechanical heart valve, atrial fibrillation, or a low risk of thromboembolism stop anticoagulants therapy 3 days before the procedure, and resume it 24 h after surgery.
(3) In moderate- to high-risk patients who are receiving acetylsalicylic acid, this is maintained around the time of surgery.
(4) For patients with a coronary stent, antiplatelet therapy is continued perioperatively.

### 2.6. Definition of Outcomes

Although surgical outcomes were reported as morbidity or mortality rates in the past, more recent studies have pointed out the appropriateness of considering them more broadly, that is, the complication rates [11,12]. The major and minor complications were defined (Table 1) based on previous reports of complications related to such devices [13,14]. On the one hand, major complications were those that placed the patient at significant risk, such as reoperation, readmissions for management, or the death of the patient. On the other hand, minor complications were those associated with patient discomfort, treated on an outpatient basis, or spontaneously resolved, with the intention of our results being compared with some other studies.

The data were prospectively collected in a registry designed for this purpose, including the basal measurements and the outcomes described.

### 2.7. Statistical Analysis

Standard descriptive statistics were used to summarize the data. Continuous variables were reported with means and SDs. The qualitative variables were measured and analyzed by proportions. The differences between groups were analyzed by Chi-square or Student-Fischer-t for qualitative or quantitative variables, respectively. The relationship between the quantitative and qualitative variables was carried out by using the Student’s t test for independent samples and the Mann–Whitney U test, respectively, depending on whether or not the normal distribution was followed.

A level of significance of 5% (*p* < 0.05) was considered in all the hypothesis verifications. The data were analyzed with the IBM SPSS version 19 (IBM, Armonk, NY, USA).

All subjects gave their informed consent for inclusion before they participated in the study. The study was conducted in accordance with the Declaration of Helsinki, and the protocol was approved by the Ethics Committee of Virgen del Rocío University Hospital, Seville, Spain (Project identification code: 2013PI/152).

## 3. Results

### Main Results

First of all, the main clinical and biological characteristics of the cases included are described in Table 2. The patients’ mean age was 76.88 ± 9.71 years, and 56.13% of the cases analyzed were male patients. The diagnosis for the pacemaker implant was mainly due to the alteration of atrioventricular conduction (49%) and sinus node disease (41.6%). These percentages differed between the two groups considered. While the most frequent diagnosis in the non-exposed group was the alteration of atrioventricular conduction (61.97%), in the exposed group the two causes had similar rates (45.19% and 46.44%).

The order in which cardiovascular risk factors were present in the cases analyzed was hypertension, obesity, dyslipidemia, diabetes, and, finally, smoking.

As a whole, the biological characteristics of the participants showed a significant baseline situation of greater fragility in the group with antithrombotic therapy, due to higher comorbidity. The characteristics of the procedure are shown in Table 3. The implant procedure shows that the central venous access was the subclavian vein (99%) and the mean duration of the procedure was almost 37 min. Most devices were located subcutaneously and the number of attempts for venous access was less than three. More dual-chamber pacemakers were implanted in the non-antithrombotic therapy group (73.24%) vs. the group with antithrombotic therapy (55.23%). In relation to the surgeon’s experience, the “non-exposed” group had a higher proportion of intervention by surgeons with much experience (63.38% vs. 46.03%, respectively).

According to Table 4, major complications occurred in 17.42% of the cases, and 10 of the patients experienced more than one major complication. The most frequent major complication in the first post-implant 24 h was pneumothorax (3.87%) and one patient showed more than one major complication in the exposed group.

During the 6-month follow-up, the complication rates in accordance with their incidence were lead dislodgement (8.39%—atrium lead in 14 patients and ventricular lead in 12 patients), followed by deceased patients (5.16%). All the deaths were located in the exposed group. In this group, nine patients experienced more than one major complication.

As a whole, the major complications were 11% more frequent in patients undergoing treatment with some type of antithrombotic therapy. This could be due to the profile of a higher baseline risk, age, and comorbidity in the “exposed” group. If deaths were excluded in the comparison, there were no differences between the two groups.

Minor complications occurred in 37.74% of the cases and there were 21 patients with more than one minor complication (Table 5).

The most frequent minor complications were phlebitis in the first 24 h (40 patients—12.90%) and hematomas (70 patients—22.58%) during the 6-month follow-up. In this last case, this minor complication was more frequent in the “exposed” group. The second most recurrent minor complication was painful shoulder (58 patients—18.71%), with a large percentage in the “non-exposed” group. It should be noted that the rates of the other minor complications identified were similar in the two cohorts.

## 4. Discussion

The present prospective study describes the complications experienced by patients who undergo permanent pacemaker implant, during the perioperative period and a follow-up of up to 6 months. The incidence of these complications between patients treated with antithrombotic therapy and those not exposed to such therapy is compared.

The incidence of complications was higher in patients with antithrombotic therapy (56%) compared with patients not exposed (52%).

### 4.1. Major Complications

A cumulative incidence of 70 major complications (22.58%) was observed in 54 patients. Patients in the exposed group presented more complications than those in the “non-exposed” group (14.08% vs. 25.10%), respectively. Previous prospective studies reported major complications oscillating between a global rate of 4.2%, reported by Tobin et al. [7] in 1332 patients, and 12.6%, reported by Parsonnet et al. [8] in 632 patients. More recent papers [17,18,19,20] reported even lower complication rates, ranging from 4% to 8%.

The most frequent major complication in the follow-up was lead dislodgement (8.39%). In our series, the rate of lead dislodgement (active fixation) was higher than the previous evidence.

Particularly, the rate of atrium-lead (7.61%) was higher than the rate identified by the literature (3% [19]–5% [18]).

Prospective studies [17,18,19] reported a lead dislodgement rate between 2% and 6%, while in a retrospective study [21] the lead dislodgement rate was 4.8%.

Information about the causes of lead dislodgement is scarce and it is often difficult to relate lead displacements to a specific etiology. Among them, it could be highlighted (a) the implant of devices in a more elderly population with greater co-morbidities, such as left ventricular dysfunction, right ventricular dilatation, and tricuspid regurgitation [19], and (b) the surgeon’s experience could be related to dislodgement rates, which suggests that inadequate initial positioning, allowance of lead slack, and/or anchoring are significant risk factors [19].

A similar problem may occur when the device is not sutured to the underlying pectoralis fascia. In this scenario, a device lying in the subcutaneous tissue (or in a submuscular space) may gradually descend through this space and exert traction on the lead.

The risk of this complication is lessened by ensuring a stable position when implanting, leaving a proper amount of intravascular lead slack so that tension is not exerted at the tip by respiration or arm motion, adequately anchoring the suture sleeve to the underlying tissue, and limiting the abduction and elevation of the ipsilateral upper extremity for a short time after implantation.

Pneumothorax was the second most frequent complication (3.87%) and usually appeared in the first 24 h after the implant. All these patients required drainage and this increased their hospital stay by two days. There were no significant differences between the cohorts. Its incidence was higher than the average rate (2%) [13,17,21,22,23], and the standard proposed by societies such as The National Cardiovascular Data Registry ICD Registry [23] and the Spanish Society of Intensive and Critical Medicine, and Coronary Units [24], which suggest a pneumothorax percentage around 0.5% and <2%, respectively, as a quality indicator. This increase, in our series, could be explained by the technique used (venous access via subclavian vein puncture) for the implant. Despite this negative impact, this method is the one preferred by more experienced surgeons. Another explanation of the reason behind the increase of the rate is the lack of use of safety measures, such as image support or the non-use of veins and alternative techniques, for example the dissection of the cephalic vein or channeling the axillary vein to insert the leads. This would reduce the incidence of pneumothorax [23,25,26,27]. The axillary vein approach seems to be a favorable technique not only for the prevention of acute complications but also to reduce lead failure, including lead insulation and lead fracture prevention, having a consequently better long-term lead survival compared with the classical subclavian approach [26].

The use of a guided image, as recommended by the current venous access guidelines [28], would be a safe practice that would avoid exposing patients to a greater risk, hence guaranteeing their safety.

An infection due to the implant was experienced in 1.61% of the patients. In this case, infection was defined as a complication that requires intravenous antibiotics and or system removal/extraction. All of them were from the exposed cohort. Our series included an infection rate, in line with previous publications, ranging between 0.4% and 13% [19,20,29]. According to previous evidence [30], this kind of infection can appear even more than 2 years after the implant, so this rate could reflect an increase if the follow-up period is extended. A less experienced surgeon could be associated with a higher probability of patients developing an infection due to the procedure. The greater length of the procedure, lower skills in surgical techniques, and longer time of prosthesis exposure increase the likelihood of contamination and more hematomas due to the increased handling of tissues.

The accumulated incidence of deceased patients was 5.16% during the 6-month follow-up, all located in the with antithrombotic therapy group (0% vs. 6.7%, *p* = 0.025). These results are similar to those reported in the literature consulted with follow-ups from 6 months to 1 year [25]. These data could be explained by the higher comorbidity present in the with antithrombotic therapy cohort and are concordant with the studies consulted [19,25], which confirm that the comorbidity of patients is a determinant factor of mortality after pacemaker implant and is related to device implant in a very low proportion. This considers the opportunity of using predictive scales based on comorbidity that could help in making complex decisions, such as limiting the therapeutic effort or pacemaker indication.

Cardiac perforation, although relatively uncommon, is a potentially life-threatening complication [31]. Clinical manifestations are variable, including cardiac tamponade, chest pain, diaphragmatic stimulation, abdominal pain, and syncope (because of pacing failure). In our study, a perforation was observed in one patient, with hemodynamic instability. However, it should be recognized that the statistics of our research are limited because of the low number of events. Although active fixation ventricular leads have been considered more prone to perforation [31], in our study, cardiac perforation used temporary pacemaker leads because they are stiffer [31].

### 4.2. Minor Complications

An accumulated incidence of 180 minor complications was observed in 117 patients, without there being significant differences between the two cohorts. Previous research [13,14,18] reported from 13% [18] to 4% [13,14] of minor complications, although comparisons are complex due to the differences in the population included in the sample [13,14].

Pocket hematomas without clinical repercussion were the most common complication (22.58%) in the 6-month follow-up. The rates were higher in the antithrombotic therapy group, although the differences are not significant. All were conservatively resolved.

The presence of hematomas in our series (22.58%) was higher than that described in previous studies (2.9% [18], 4% [17], 5% [20]).

These differences could be explained by the inclusion criteria of patients, the periods of suspension of antithrombotic therapy, the type of implanted devices, the definition of hematoma, or by the study design. The periods of suspending antithrombotic therapy could be explained by the disorder caused by discontinuation/re-initiation and the effect of the combination of different drugs on the coagulation cascade, causing a new bleed that is not controlled during the intraoperative stage. Nevertheless, if anticoagulation is still active, bleeding can be better controlled, allowing local hemostatic measures to be taken.

It would be convenient to prepare recommendations about those strategies that are currently demonstrating higher safety, such as maintaining oral anticoagulation during pacemaker implantation in patients with a high thrombotic risk [11]. This new strategy would lead us to an optimization of measures and care for the prevention of hemorrhagic complications (a careful evaluation of the wound before discharge and informing patients and their relatives about warning signs), without an increase in the risk of thrombosis [32].

The painful shoulder ipsilateral to the pacemaker implant is a complication usually overlooked and can be disabling for individuals affected [33]. The accumulated incidence of painful shoulder was 18.71%, significantly more frequent in the cohort of not-exposed patients. It seems possible that these results are due to the number of days the patients had their arms immobilized, and that this was longer in the not-exposed group. This finding supports previous research [33,34,35], which reports 62% of shoulder pain, 42% with tendinitis 3 months after implantation [34], and 1.1% of frozen shoulder and 33% of shoulder pain [35]. All these data would confirm the relationship between the immobilization of the arm and the presence of painful shoulder [33]. Such results differ from ours due to the inclusion of larger devices (defibrillators and resynchronizers), which would explain higher percentages of this complication.

Therefore, updating and personalizing the information and the recommendations given to each patient regarding the immobilization of their arms would guarantee the safety of the care offered. In those people who need more time of immobilization, it would be appropriate to increase their follow-up, by telephone or by means of an ambulatory face-to face consultation, developing a subsequent rehabilitation plan that would minimize the effects of this measure.

### 4.3. Limitations

This is a single center study and, therefore, its outcomes might not necessarily be generalizable regarding the number of complications, though it would be regarding their causes. The level of experience of the perioperative nursing team has not been included in the study and therefore its impact on the final outcome is unknown.

## 5. Conclusions

In conclusion, patients who were implanted with a permanent pacemaker in our center in 2014 were mostly men, predominantly elderly, with more cardiovascular risk factors, and a higher comorbidity index in the group with antithrombotic therapy, which mark them as a more fragile group.

There was a relevant rate of complications related to a permanent pacemaker implant. These complications occurred in the initial phase after the implant and decreased during the follow-up, the most frequent ones being pneumothorax, lead dislodgement, peripheral phlebitis, uncomplicated hematomas, and painful shoulders.

Adverse event variables should be introduced in future pacemaker implant registration reports to enrich our knowledge concerning the quality of the implant, in accordance with the current safety culture, as well as to use them to improve the practice.

## Figures and Tables

**Table 1:** Major and minor complications.

Major Complications	Minor Complications
Cardiac perforation/cardiac tamponade	Cellulitis
Death	Local pain
Generator or lead malfunction (lead break, bad connection lead-generator)	Shoulder pain
Hematomas with a clinical significance	Peripheral nerve injury
Infection	Superficial phlebitis
Lead dislodgement	Uncomplicated hematomas
Pneumothorax/hemothorax	
Pre-erosion or erosion of pocket	
Thromboembolic event (transient ischemic attack, Stroke, pulmonary thromboembolism, thrombosis, deep venous thrombosis)	

**Table 2:** Pre-implant clinical and biological values characteristics.

		Total *n* = 310 (%)	Non-Exposed *n* = 71 (%)	Exposed *n* = 239 (%)	*p*-Value
Age (mean ± SD)		76.88 ± 9.71	75.25 ± 12.95	77.36 ± 8.48	NS
Gender	Male	174 (56.13%)	38 (53.52%)	136 (56.90%)	NS
	Female	136 (43.87%)	33 (46.48%)	103 (43.10%)	
INR (Mean ± SD)		1.10 ± 0.19	1.04 ± 0.09	1.12 ± 0.21	0.000
Diagnosis for intervention	Sinus node disease	129 (41.6%)	18 (25.35%)	111 (46.44%)	0.006
	AV conduction system disease	152 (49%)	44 (61.97%)	108 (45.19%)	
	Syncope and others	29 (9.4%)	9 (12.68%)	20 (8.37%)	
Cardiovascular risk factors	Hypertension	237 (76.45%)	46 (64.79%)	191 (79.92%)	0.008
	Diabetes	109 (35.16%)	15 (21.53%)	94 (39.33%)	0.004
	Dyslipidemia	134 (43.23%)	23 (32.39%)	111 (46.44%)	0.035
	Obesity (BMI > 28)	186 (60%)	37 (52.11%)	149 (62.34%)	NS
	Smoking	28 (9.03%)	9 (12.68%)	19 (7.95%)	NS
Charlson score [15]	Absence of comorbidity	202 (65.16%)	59 (83.10%)	143 (59.83%)	-
	Low and high comorbidity	108 (34.84%)	12 (16.90%)	96 (40.17%)	
HAS-BLED score [16]	Low	96 (30.97%)	48 (67.61%)	48 (20.08%)	
	Medium	153 (49.35%)	22 (30.99%)	131 (54.81%)	<0.000
	High	61 (19.68%)	1 (1.41%)	60 (25.10%)	
Venous thrombotic risk [9]	Low	108 (34.84%)	40 (56.34%)	68 (28.45%)	0.000
	Medium	60 (19.35%)	16 (22.54%)	44 (18.41%)	NS
	High	142 (45.81%)	15 (21.13%)	127 (53.14%)	0.000

AV: Atrioventricular, BMI: Body Mass Index, DVT: Deep Venous Thrombosis, HAS-BLED: Hypertension, Abnormal Renal/Liver Function, Stroke, Bleeding History or Predisposition, Labile INR, Elderly, Drugs/Alcohol Concomitantly, INR: International Normalized Ratio, *n*: number of patients, NS: Not Significant, SD: Standard Deviation.

**Table 3:** Implant-related characteristics.

		Total *n* = 310 (%)	Non-Exposed *n* = 71 (%)	Exposed *n* = 239 (%)	*p*-Value
Cauterizer		17 (5.48%)	4 (5.63%)	13 (5.44%)	NS
Incision prior to venous puncture		97 (31.29%)	28 (39.44%)	69 (28.87%)	NS
Subclavian vein access ^1^		308 (100%)	70 (98.59%)	238 (99.58%)	NS
Arterial puncture		48 (15.48%)	9 (12.68%)	39 (16.32%)	NS
Temporary pacemaker		34 (10.97%)	5 (7.04%)	29 (12.13%)	NS
Number of attempts venous access	<3	229 (73.87%)	54 (76.06%)	175 (73.22%)	NS
	>3	74 (23.87%)	15 (21.13%)	59 (24.69%)	
	The opposite side	7 (2.26%)	2 (2.82%)	5 (2.09%)	
Device type	Pacemaker, dual	184 (59.35%)	52 (73.24%)	132 (55.23%)	0.006
	Pacemaker, single	126 (40.65%)	19 (26.76%)	107 (44.77%)	
Device location	Subcutaneous	299 (96.45%)	68 (95.77%)	231 (96.65%)	NS
	Subpectoral	11 (3.55%)	3 (4.23%)	8 (3.35%)	
Surgeon experience	Low	61 (19.68%)	11 (15.49%)	50 (20.92%)	NS
	Medium	94 (30.32%)	15 (21.13%)	79 (33.05%)	0.054
	High	155 (50%)	45 (63.38%)	110 (46.03%)	0.010
Duration of implantation (Mean ± SD)		36.99 ± 15.47	37.66 ± 15.19	36.79 ± 15.50	NS

^1^ Two patients are not included in the venous access, this being considered as anecdotal (one is accessed by a cephalic vein and another by a femoral vein). *n*: number of patients, NS: Not Significant, OAC/NOAC: Oral Anticoagulation/New Oral Anticoagulation, SD: Standard Deviation.

**Table 4:** Major complications are shown as periprocedural or subsequent up to 6 months of the follow-up.

**Periprocedural (24 h)**	**Total** ***n* = 310**	**Non-Exposed** ***n* = 71**	**Exposed** ***n* = 239**	***p*-Value**
Pneumothorax	12 (3.87%)	3 (4.23%)	9 (3.76%)	NS
Cardiac perforation	1 (0.32%)	0	1 (0.42%)	NS
Cardiac tamponade	2 (0.64%)	0	2 (0.84%)	NS
Total patients with >1 major complication	1 (0.32%)	0	1 (0.42%)	NS
**Subsequent Up to 6 Months**	**Total** ***n* = 310**	**Non-Exposed** ***n* = 71**	**Exposed** ***n* = 239**	***p*-Value**
Lead dislodgement	26 (8.39%)	6 (8.45%)	20 (8.37%)	NS
Malfunction	2 (0.64%)	1 (1.41%)	1 (0.42%)	NS
Pre-erosion or erosion of pocket	1 (0.32%)	0	1 (0.42%)	NS
Infection	5 (1.61%)	0	5 (2.09%)	NS
Stroke	4 (1.29%)	0	4 (1.67%)	NS
Death	16 (5.16%)	0	16 (6.69%)	0.025
Hematomas with clinical significance	1 (0.32%)	0	1 (0.42%)	NS
Total patients with >1 major complication	9 (2.90%)	0	9 (3.76%)	0.097

*n*: number of patients, NS: Not Significant.

**Table 5:** Minor complications are shown as periprocedural or subsequent up to 6 months of the follow-up.

**Periprocedural (24 h)**	**Total** ***n* = 310**	**Non-Exposed** ***n* = 71**	**Exposed** ***n* = 239**	***p*-Value**
Superficial phlebitis	40 (12.90%)	9 (12.68%)	31 (12.97%)	0.948
Total patients with >1 minor complication	0	0	0	
**Subsequent Up to 6 Months**	**Total** ***n* = 310**	**Non-Exposed** ***n* = 71**	**Exposed** ***n* = 239**	***p*-Value**
Uncomplicated hematomas	70 (22.58%)	11 (15.49%)	59 (24.69%)	NS
Peripheral nerve injury	5 (1.61%)	1 (1.41%)	4 (1.67%)	NS
Pain shoulder	58 (18.71%)	20 (28.17%)	38 (15.90%)	0.019
Cellulitis	1 (0.32%)	0	1 (0.42%)	NS
Local pain	6 (1.93%)	1 (1.41%)	5 (2.09%)	NS
Total patients with >1 minor complication	21 (6.77%)	6 (8.45%)	15 (6.27%)	NS

*n*: number of patients, NS: Not Significant.
